# EEG analysis in patients with schizophrenia based on microstate semantic modeling method

**DOI:** 10.3389/fnhum.2024.1372985

**Published:** 2024-04-04

**Authors:** Hongwei Li, Changming Wang, Lin Ma, Cong Xu, Haifeng Li

**Affiliations:** ^1^Faculty of Computing, Harbin Institute of Technology, Harbin, China; ^2^Department of Neurosurgery, XuanWu Hospital, Capital Medical University, Beijing, China

**Keywords:** schizophrenia, microstate analysis, semantic features, quality features, dual-microstate templates

## Abstract

**Introduction:**

Microstate analysis enables the characterization of quasi-stable scalp potential fields on a sub-second timescale, preserving the temporal dynamics of EEG and spatial information of scalp potential distributions. Owing to its capacity to provide comprehensive pathological insights, it has been widely applied in the investigation of schizophrenia (SCZ). Nevertheless, previous research has primarily concentrated on differences in individual microstate temporal characteristics, neglecting potential distinctions in microstate semantic sequences and not fully considering the issue of the universality of microstate templates between SCZ patients and healthy individuals.

**Methods:**

This study introduced a microstate semantic modeling analysis method aimed at schizophrenia recognition. Firstly, microstate templates corresponding to both SCZ patients and healthy individuals were extracted from resting-state EEG data. The introduction of a dual-template strategy makes a difference in the quality of microstate sequences. Quality features of microstate sequences were then extracted from four dimensions: Correlation, Explanation, Residual, and Dispersion. Subsequently, the concept of microstate semantic features was proposed, decomposing the microstate sequence into continuous sub-sequences. Specific semantic sub-sequences were identified by comparing the time parameters of sub-sequences.

**Results:**

The SCZ recognition test was performed on the public dataset for both the quality features and semantic features of microstate sequences, yielding an impressive accuracy of 97.2%. Furthermore, cross-subject experimental validation was conducted, demonstrating that the method proposed in this paper achieves a recognition rate of 96.4% between different subjects.

**Discussion:**

This research offers valuable insights for the clinical diagnosis of schizophrenia. In the future, further studies will seek to augment the sample size to enhance the effectiveness and reliability of this method.

## 1 Introduction

Schizophrenia (SCZ) is a severe and debilitating mental disorder, the etiology and pathogenesis of which remain incompletely understood (Sekar et al., 2016). Electroencephalography (EEG) captures dynamic changes in electrical signals within the cerebral cortex, providing insights into brain functional states (Barros et al., 2021). Due to its non-invasive nature, high temporal resolution, cost-effectiveness, and the ability for continuous monitoring, EEG holds a significant position in the fields of neuroscience and the diagnosis of neurological disorders (Hamilton and Northoff, 2021). Investigating EEG features closely associated with schizophrenia is of paramount significance as it can deepen our comprehension of its etiology and enhance the accuracy of SCZ diagnosis.

Existing research in cognitive neuroscience has demonstrated the existence of electrical microstates in the brain. Scalp voltage distribution maintains a semi-stable state within a brief temporal window (typically 80–150 ms). This means that the topological structure of brain maps remains relatively stable for a certain duration, then rapidly transitions to another state before stabilizing again, displaying discontinuity. During the stable state, the strength of the scalp potential may increase or decrease, but the topography remains stable (Lehmann et al., 1987; Pascual-Marqui et al., 1995; Khanna et al., 2014). To investigate the evolving overall functional patterns of the brain across time, Lehmann and his colleagues introduced the concept of Microstate. They defined four microstates (labeled A, B, C, and D), corresponding to resting-state networks associated with the auditory network, visual network, salience network, and attention network in the brain. They transformed the raw EEG data into a sequence of these four states that alternate (Lehmann et al., 1987). Scholars usually categorize resting-state EEG waves into four microstates based on their topological structures. These microstates are: right frontal-left posterior (labeled as A), left frontal-right posterior (labeled as B), midline frontal-occipital (labeled as C), and midline frontal (labeled as D). Michel and Koenig (2018) discussed in their review of the current and future directions of microstate analysis that these microstates account for 65–80% of the original data's features. These brain maps exhibit high similarity across healthy individuals, patients with various conditions, and distinct mental states, such as rest and sleep (Koenig et al., 2002). Microstate analysis has the capability to characterize quasi-stable scalp potential at a sub-second scale while preserving the temporal dynamics of EEG and the spatial information of scalp potential distribution. This method represents an innovative approach to quantifying brain electrical signals with potential neurophysiological significance (Khanna et al., 2014).

Microstate sequences are widely employed in the investigation of SCZ due to their rich pathological and semantic information (Lehmann et al., 2005). Research indicates that different psychological states and thought categories may have underlying correlations with different microstate topologies. Therefore, researchers consider microstates as “thought atoms,” the basic units constituting emotions and cognition (Lehmann et al., 1998). Normal and abnormal cognitive states may manifest varying patterns in microstate sequences. Based on this conceptual framework, the frequency, duration, and sequence patterns of microstates can be used to explain the occurrence of psychological abnormalities and specific behavioral symptoms in individuals with SCZ, thereby mapping their cognitive states within a relatively brief time frame (Yan et al., 2023). Among the four microstate classes, the temporal dynamics of Microstate C and D are regarded as potential endophenotypes of SCZ (Chang et al., 2022; Lin et al., 2022; Chen P.-H. et al., 2023). Researchers have found that, compared to the control group, SCZ patients exhibit continuous increases in the time coverage and occurrence rate of Microstate C, while the time coverage and average duration of Microstate D significantly decrease (da Cruz et al., 2020). Studies also utilize source localization algorithms to investigate cortical layer activations corresponding to the topologies of microstate, revealing significant activation in the left inferior parietal lobule and left temporal gyrus in the brains of SCZ patients (Soni et al., 2018; Chen P.-H. et al., 2023). This effectively reveals the temporal dynamics of SCZ pathology. Leveraging changes in microstate parameters in SCZ patients, scholars have utilized microstates as crucial neural imaging biomarker in the automated identification of schizophrenia (Baradits et al., 2020; Luo et al., 2020; Wang et al., 2021). For instance, Baradits et al. (2020) used four microstate time parameters as features for SCZ classification and achieved 82.7% recognition accuracy. Kim et al. extracted 19 microstate features and 31 traditional EEG features from the resting-state EEG of SCZ patients, combining them with machine learning for identification. The results demonstrated that microstate features (76.62%) outperformed traditional EEG features (68.89%) in SCZ identification, displaying better classification performance. This suggests the potential of microstates as valuable neural imaging biomarkers for brain disorders, enabling a more effective representation of patients' abnormal states (Kim et al., 2021).

However, there are still some problems that need to be solved in the identification of SCZ by microstate analysis.

(1) An important consideration is the transferability of microstate templates between healthy individuals and SCZ patients, specifically, whether the same set of templates can effectively model EEG signals in both groups. At present, there is no publicly available research exploring this specific issue. Due to the similarity of microstates under different conditions, researchers typically model the EEG signals of healthy individuals and SCZ patients uniformly. Although this method can effectively reduce computational complexity, it overlooks the quality characteristics of microstate sequence.
(2) The present research is limited to analyzing the statistical characteristics of the temporal parameters of individual microstate, but does not consider the temporal parameters when the microstate combination appears. This limitation may affect the comprehensive understanding of microstate sequence.
(3) Determining the appropriate length of microstate sequences is imperative for efficient feature extraction and recognition and warrants further investigation. Sequences that are too short may not accurately capture certain microstate features, thus impacting the accuracy, while sequences that are too long may increase the computational burden and processing time.

Therefore, there is a compelling requirement for a more systematic exploration of the application of microstate analysis techniques in the identification of SCZ, along with the need to further substantiate the reliability and biological significance of the findings. This will serve to enhance the provision of enhanced clinical decision support for the prevention, treatment, and rehabilitation of SCZ.

The main contributions of this work are summarized as follows:

1. This study introduced the concept of microstate semantic features into traditional microstate research and identifies microstate semantic sequences highly correlated with SCZ.
2. The study proposed a dual-template microstate construction strategy to effectively capture differences in microstates between SCZ patients and the healthy group. Additionally, microstate quality features based on these differences were proposed.
3. The study validated the new features on the Warsaw database for SCZ recognition to assess the effectiveness of microstate semantic features and quality features. The results demonstrated that the method proposed in this paper achieves optimal SCZ recognition accuracy.

**Figure 1:**
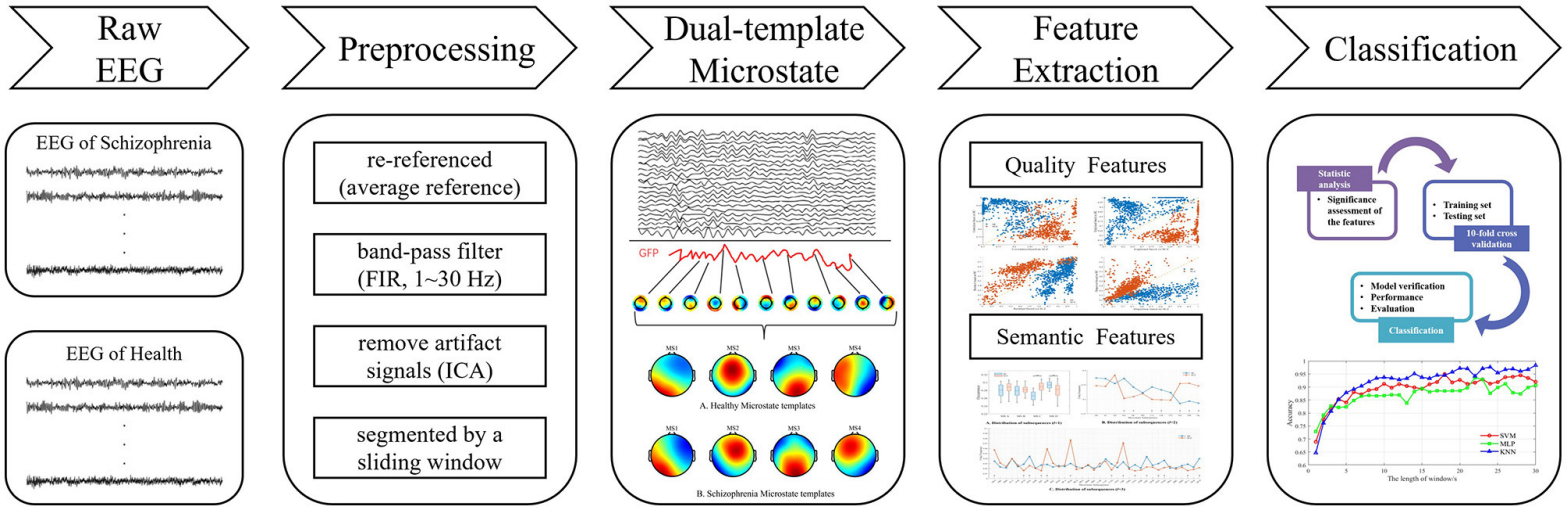
Framework of the proposed scheme for identifying SCZ patients using EEG signals.

The overall research content of this paper is illustrated in Figure 1.

The rest of this paper is organized as follows. Materials and methods are explained in Section II. Experimental results and their corresponding discussions are found in Sections III and, followed by the conclusion in Section IV.

## 2 Materials and methods

### 2.1 Dataset

We utilized a publicly available EEG dataset for our study, and the study protocol received approval from the Ethics Committee of the Institute of Psychiatry and Neurology in Warsaw (Olejarczyk and Jernajczyk, 2017). Prior to their participation, all individuals received a written explanation of the study protocol and provided written consent. The dataset comprised data from 14 patients diagnosed with schizophrenia and 14 healthy control subjects. The patients' group consisted of seven males (with an average age of 27.9 ± 3.3 years) and seven females (with an average age of 28.3 ± 4.1 years) who were diagnosed with paranoid schizophrenia in accordance with the International Classification of Diseases (ICD)-10-CM criteria (F20.0) and exhibited pronounced positive symptoms. Inclusion criteria also necessitated a washout period of more than one week, with early-stage patients, including those experiencing their first episodes, being excluded. Exclusion criteria encompassed conditions such as pregnancy, organic brain pathology, severe neurological diseases (e.g., epilepsy, Alzheimer's, or Parkinson's disease), and the presence of a general medical condition. The patient and control groups were matched for both age and gender. EEG data were recorded using a nineteen-channel setup, adhering to the International 10/20 EEG system, at a sampling frequency of 250 Hz, during a 15-minute session of eyes-closed resting state. Further details regarding the dataset can be accessed at the repository and its corresponding article (Olejarczyk and Jernajczyk, 2017).

### 2.2 EEG data preprocessing

Standard EEG pre-processing techniques were employed to eliminate mixed noise, including electrooculograms (EOG) and electromyograms (EMG). The primary pre-processing steps included:

(1) Manual removal of obvious noises through visual inspection.
(2) Re-referencing EEG signals to the average reference.
(3) Applying a band-pass filter (FIR, 0.1–40 Hz) to EEG signals.
(4) Utilizing Independent Component Analysis (ICA) to eliminate artifact signals, such as EOG and EMG.

Following the pre-processing, EEG signals were segmented using a sliding window approach. Depending on the length of the sliding window, the original dataset was transformed into various new datasets. Detailed information about data segmentation will be presented in the experimental section.

### 2.3 Microstate model analysis

The microstate model analysis regards the multichannel EEG signal as a time series composed of topographies representing instantaneous potential distributions. Assuming that *V* = (*V*_1_, *V*_2_, ..*V*_*t*_, ...*V*_*T*_, ) denotes a segment of *N*-channel EEG signals of duration *T*, where $V\in R^{N\times T},V_{t}\in R^{N\times 1}$. Then the microstate sequence o can be constructed as follows.

(1) The Global Field Power (GFP) of the EEG was computed at each time point. The topography at the peak point of the GFP has the strongest signal strength and the highest signal-to-noise ratio, and the potential distribution at the localized peak of the GFP remains stable.


(1) $$GFP(t)=\sqrt{\frac{\sum_{i=1}^{N}{\left(v_{i}(t)-\bar{v}(t)\right)}^{2}}{N}}$$

where *N* denotes the number of electrodes, *v*_*it*_ denotes the potential value of the *i*-th electrode at time *t*, and *v*_*t*_ denotes the average value of the potential of all electrodes at time *t*.

(2) The potential topography at the GFP peak was extracted to construct the sample set. The construction process can be expressed by Equation (2).


(2) $$\begin{array}{l} S=\left\{V_{t}|1<t<T\wedge G_{t-1}<G_{t}>G_{t+1}\right\} \end{array}$$

(3) The microstate template Γ_*k*_ can be expressed in Equation (3).


(3) $$\begin{array}{l} V_{t}=\overset{K}{\underset{k=1}{\sum }}a_{kt}\Gamma_{k}+E_{t} \end{array}$$

where *K* denotes the number of microstate templates, $\Gamma_{k}\in R^{N\times 1}$ is the *k*-th microstate template, *a*_*kt*_∈{0, 1} denotes the intensity of the *k*-th microstate at moment *t*, and *E*_*t*_ is the fitting error.

(4) The microstate template Γ_*k*_ can be computed by minimizing the Equation (4).


(4) $$\begin{array}{l} loss=\frac{1}{T_{s}(N-1)}\sum_{t=1}^{T}\delta_{t}{\left\|V_{t}-\sum_{k=1}^{K}a_{kt}\Gamma_{k}\right\|}^{2}\\ \;\delta_{t}=\{\begin{array}{ll} 1, & \text{ if }V_{t}\in S\\ 0, & \text{ if }V_{t}\notin S \end{array} \end{array}$$

where *T*_*s*_ denotes the number of elements in the set *S*. The microstate template Γ_*k*_ can be obtained by solving the above equation by clustering algorithm or Lagrange multiplier.

(5) The distance between the corresponding brain topology map and the microstate template is calculated for each moment, and the brain state at that moment is labeled as the nearest template. Label allocation can be represented by Equation (5).


(5) $$L_{t}=\underset{k}{\arg\;\min}\left\{\left|V_{t}^{T}V_{t}-V_{t}^{T}\Gamma_{k}\right|\right\}$$

Up to this point, the multichannel EEG signal has been decomposed into a time sequence comprising *K* alternating microstates.

### 2.4 Dual-template microstate model

**Figure 2:**
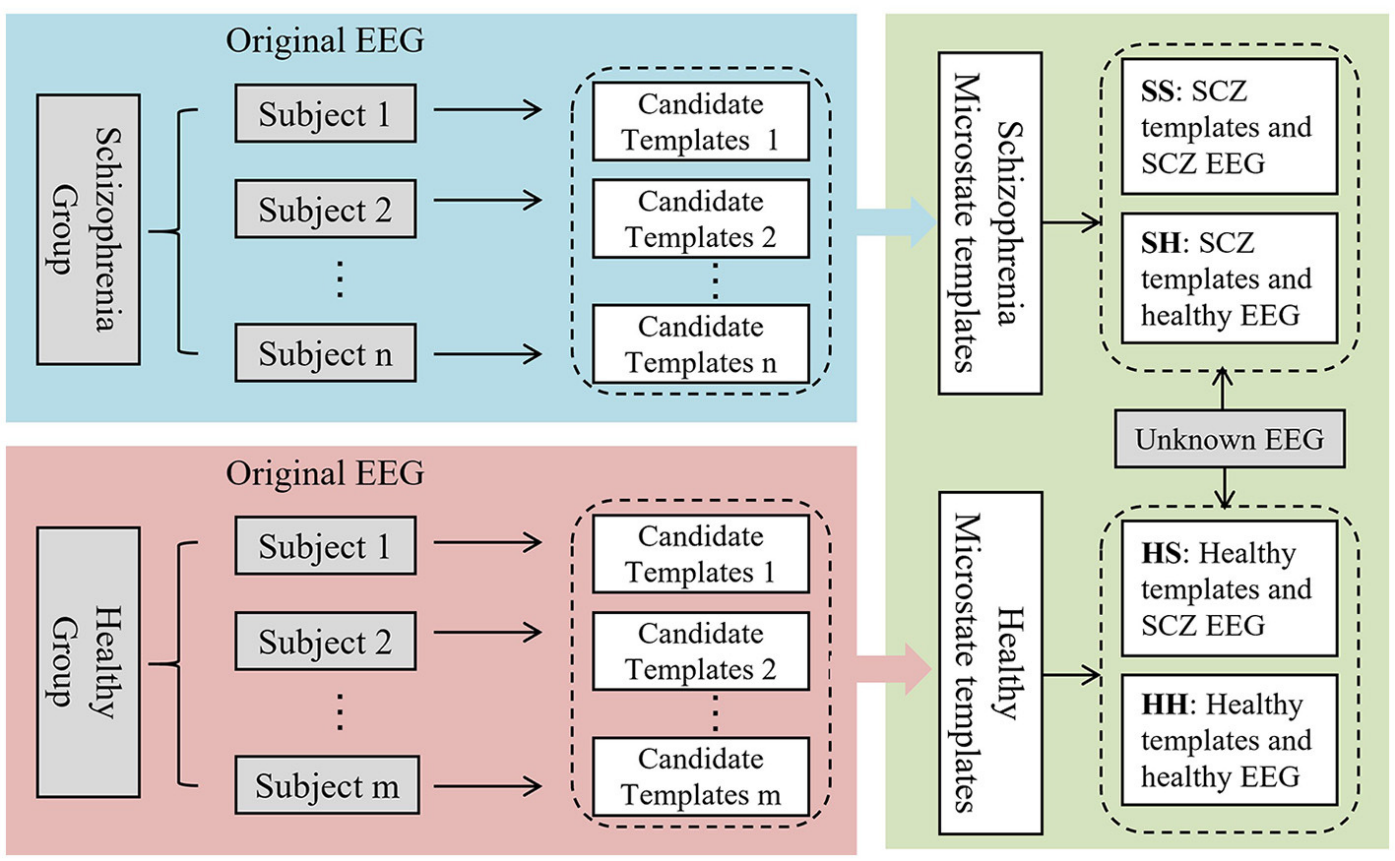
Overview of dual-template microstate modeling method. SS: The SCZ template was used to model the EEG of SCZ patients. SH: Model EEG of healthy subjects using SCZ templates. HS: EEG signals of SCZ patients were modeled using healthy subject templates. HH: Modeling EEG of healthy subject using healthy subject template.

Conventional microstate analysis usually mixes the EEG signals from the SCZ patients and the healthy individuals to generate a set of microstate templates for subsequent analysis and feature extraction. However, according to neuroimaging studies, SCZ patients exhibit significant difference in brain structure and function, which may result in different topographies of scalp potential. Therefore, employing the same template for modeling both datasets may overlook these differences. In response to this concern, this paper introduces a novel approach, the dual-template microstate model method, designed to more accurately capture the microstate differences between SCZ patients and healthy subjects. The workflow of the dual-template microstate modeling approach is illustrated in Figure 2.

(1) Conduct clustering for each individual and extract the microstate template as a candidate template. In this process, DBSCAN clustering algorithm was employed. DBSCAN is a density-based clustering algorithm known for its ability to cluster dense datasets with arbitrary shapes and its resilience to anomalies within the dataset. It effectively avoids the impact of singularities in the EEG signal on the clustering outcomes. The steps are outlined as follows:

a. According to Equation (1), Calculate the global field power (GFP) of the subject at each time point, and extract the topography corresponding to the GFP peak to construct a set of topographies.
b. Initialize the parameters of the DBSCAN clustering algorithm, including radius *r* and density threshold minPts. The radius *r* defines the size of the neighborhood around the sample, while the density threshold minPts determines the minimum number of samples required to form a set. During this process, Euclidean distance was used as the distance metric.
c. For each sample in the set of topographies, calculate the number of samples in its neighborhood. If the number of samples exceeds the density threshold minPts, the sample is labeled as a core point.
d. Form a new set of each core point and all points within its neighborhood. If two sets intersect, merge them into one set.
e. If the total number of formed sets is not equal to 4, adjust *r* and minPts of the DBSCAN algorithm, and repeat steps c to e until the total number of sets reaches 4.
f. Calculate the average value of samples in each set as the microstate template.

(2) The candidate templates obtained from all subjects were divided into healthy and SCZ groups based on task-related information. The topographies within each group are subsequently re-clustered by the *K*-means algorithm. Given the absence of singularities in the candidate templates resulting from the initial clustering in step (1), and the high similarity among topographies within the same microstate class, the *K*-means algorithm is employed for rapid clustering in this stage. The steps are outlined as follows:

a. Randomly select four topographies from the health group as initial clustering centers.
b. Calculate the spatial similarity between the remaining topographies and the initial clustering centers. Assign each topography to the class associated with the clustering center exhibiting the highest spatial similarity.
c. Calculate new clustering centers based on the topographical maps within each class.
d. Repeat the procedure until the clustering centers are no longer changed, at which time the resulting four clustering centers are the microstate templates for the health group.
e. Repeat the above process to calculate the microstate templates for the SCZ group.

(3) After calculation, the healthy group and the patient group, respectively, obtained corresponding microstate templates, each template containing four microstates. Assign the most relevant microstate labels based on the spatial correlation between the topology of the EEG at each time point and the four microstates. For EEG signals with known labels, the dual-template-based microstate construction strategy can transform the original EEG signals into four types of microstate time series, as outlined in Table 1. As for the samples with unknown labels, there are two possibilities for these two microstate sequences, (1) SS and HS, and (2) SH and HH.

**Table 1:** Different microstate sequence type.

**Type**	**Meaning**
SS	Modeling the EEG of SCZ patients by the SCZ templates
SH	Modeling the EEG of healthy individuals by the SCZ templates
HS	Modeling the EEG of SCZ patients by the healthy templates
HS	Modeling the EEG of healthy individuals by the healthy templates

### 2.5 Microstate features

In this section, microstate feature extraction will be performed for these two microstate sequences, and these features will be used for the identification of SCZ in the subsequent sections. A total of 39-dimensional microstate features were extracted as inputs to the classifier in this paper, including 16-dimensional quality features, 21-dimensional semantic features, and 12-dimensional traditional temporal features.

#### 2.5.1 Microstate temporal features

In this paper, the traditional temporal parameters (Lin et al., 2023) were extracted on two microstate sequences as temporal features of the sequences, including:

(1) Mean duration (MD): mean duration (in ms) is the average time that a given microstate was uninterruptedly present.
(2) Occurrence per second (OPS): occurrence is the mean number of times a given microstate is occurring per second.
(3) Time coverage ratio (TCR): time coverage (in %) is the percentage of the total analysis time spent in a given microstate

#### 2.5.2 Microstate semantic features

Current research on microstates regards individual microstates as isolated states and focuses on the association of parameters such as occurrence and duration of individual states with mental illness. However, this approach may not adequately capture the dynamic evolution and interactions between microstates and analyze the sequential patterns, trends, or periodicity of microstates, which could overlook important information embedded in the time series. Therefore, this study introduced the concept of semantic characterization of microstates. This approach provides new possibilities for exploring the association between microstates and SCZ by decomposing microstate sequences into subsequences, and extracting subsequences imbued with specific semantics through a comparative analysis of diverse time parameter statistics among various subsequences. The microstate sequence encapsulates a wealth of physiological and pathological information, intricately mirrored by the variability and randomness inherent in different states and subsequences within the microstate sequence, which is the theoretical basis for using this method.

Given a microstate sequence of length *N*_*s*_, the sequence contains a total of *K* kinds of microstate. For this microstate sequence, there are a total of *K*^*l*^ kinds of subsequences of length *l*. For this sequence, the following features were extracted:

(1) Subsequence frequency: the frequency of occurrence of a subsequence with length *l* in the microstate sequence is calculated as shown in the Equation (6).


(6) $$f_{i}=\frac{Occ_{i}}{N_{s}-l+1},i\in \left[1,K^{l}\right]$$

where *Occ*_*i*_ denotes the number of times the subsequence appears in the whole sequence.

(2) Average duration of subsequence: the average duration of a subsequence with length l in a microstate sequence is calculated as shown in the Equation (7).


(7) $$d_{i}=\frac{Tt_{i}}{l\times Occ_{i}},i\in \left[1,K^{l}\right]$$

where *Tt*_*i*_ denotes the length of time that the subsequence appears in the whole sequence.

Computing temporal parameters for subsequences of different lengths achieves a deeper understanding of the relationship between microstates and SCZ. The introduction of this approach is expected to provide additional insights into microstate analysis and enhance the methodology and theory in the field of SCZ research.

#### 2.5.3 Microstate quality features

As mentioned earlier, SCZ patients exhibit substantial structural and functional alterations in the brain, which can result in variations in the topography of potential distribution on the scalp surface. Consequently, differences in data expressiveness and spatial correlation are observed in microstate sequences modeled by different microstate templates. In other words, the fitting quality of SS (same class) and HH (same class) sequences should be significantly higher than that of SH (different class) and HS (different class) sequences.

Therefore, in this paper, the quality of two sequences were evaluated in terms of spatial correlation, data explanatory, residual and dispersion. The ratio of the quality parameters between the two sequences was extracted as microstate quality feature.

(1) Spatial correlation: the average spatial correlation of all potential topographies in each class of microstates with the microstate template. This feature is calculated as Equation (8).


(8) $$msc=\sum_{t=1}^{T}\frac{\left|V_{t}^{T}\Gamma_{t}\right|}{\left\|V_{t}||||\Gamma_{t}\right\|}$$

where Γ_*t*_ denotes the microstate template assigned to the EEG signal at time *t*, and ||*a*|| denotes the Euclidean norm of the vector *a*.

(2) Global explanatory variance: this parameter is used to measure the percentage of data that can be explained by microstate classes. The more accurate the microstate template, the higher the value of GEV. The calculation of this feature is shown as Equation (9).


(9) $$gev=\frac{\sum_{i=1}^{T}{\left(G_{t}\times \frac{\left|V_{t}^{T}\Gamma_{t}\right|}{\left\|V_{t}||||\Gamma_{t}\right\|}\right)}^{2}}{\sum_{t^{\prime }=1}^{T}G_{t}}$$

(3) Residual: this parameter is used to estimate the residual margin after transforming the EEG signal into a microstate sequence. The more accurate the microstate template, the lower the value of the residual margin. The Equation (10) is the calculation method for this feature.


(10) $$err=\frac{\sum_{t=1}^{T}\left(V_{t}^{T}V_{t}-{\left(\Gamma_{t}^{T}V_{t}\right)}^{2}\right)}{NT}$$

(4) Dispersion: this parameter is a measure of the average distance between members of the same class. It is calculated as Equation (11).


(11) $$w=\sum_{k=1}^{K}\frac{\sum_{V_{t}\in S_{k}}\sum_{V_{t^{\prime }\in S_{k}}}{\left\|V_{t}-V_{t^{\prime }}\right\|}^{2}}{T_{k}}$$

where *S*_*k*_ denotes the set of EEG data labeled as microstate *k*, and Γ_*k*_ denotes the number of elements in the set *S*_*k*_.

## 3 Experiment and results

### 3.1 Microstate quality analysis

**Figure 3:**
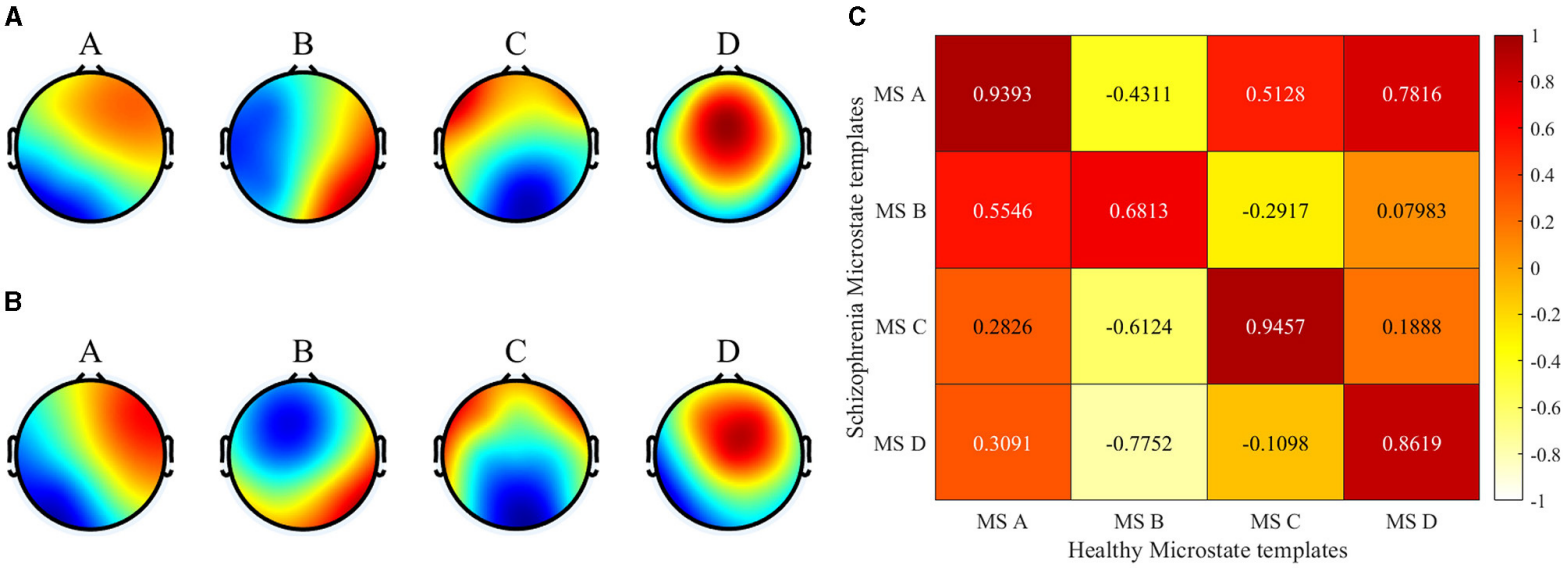
Microstate clustering results. **(A)** Healthy templates from 14 healthy subjects. **(B)** SCZ templates from 14 patients with schizophrenia. **(C)** Spatial correlation between healthy templates and SCZ templates).

The brain topography resulting from microstate clustering is shown in Figure 3. The figure illustrates that the microstate distributions of the SCZ patients and the healthy individuals (Healthy Control, HC) exhibit a general similarity, with localized differences in microstates B and D.

This article evaluates the quality of microstate sequences from four aspects: spatial correlation, data expression ability, residual margin, and dispersion. The average quality evaluation results of the four microstate sequences are shown in Table 2.

**Table 2:** Average quality assessment results of microstate sequences.

**Parameters**	**HH**	**SS**	**HS**	**SH**
Correlation	92.8%	93.9%	80.6%	82.1%
Explained	85.5%	88.5%	74.4%	78.9%
Residual	0.63	0.47	0.87	0.85
Dispersion	0.22	0.21	0.45	0.34

According to the table, it can be observed that in the combination of template and data consistency, the GEV of SCZ patients reached 88.5%, while the GEV of HC was 85.5%. This indicates that the two microstate templates can effectively express the corresponding original brain topographic. However, in inconsistent combinations, the average GEV of the HS sequence was only 74.4%, while the average GEV of the SH sequence was 78.9%. This means that inconsistent combinations exhibit significantly lower quality while expressing the original data. Other quality evaluation indicators have also produced similar results, emphasizing that the combination of template and data consistency exhibits higher quality in constructing microstate sequences. These results have important implications for the accuracy and reliability of microstate analysis.

**Figure 4:**
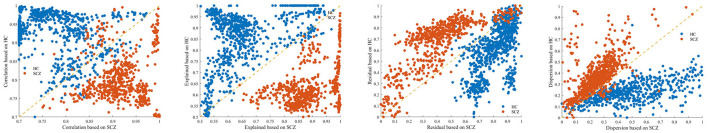
Distribution of data series quality features. From left to right: correlation, explained, residual, dispersion; blue points in the figure indicate HC data, red points indicate SCZ data.

In order to deeply evaluate the role of the quality features in the identification of SCZ, this paper plots the distribution of data under different quality features by taking the quality features of microstate sequences constructed by SCZ templates as the horizontal coordinates and the quality features of microstate sequences constructed by HC templates as the vertical coordinates. The specific results are shown in Figure 4. This distribution plot helps us to understand the influence of different quality features on SCZ identification and provides an important reference for further research.

From the figure, it can be clearly observed that the quality features are significantly discriminative in the identification of SCZ. In terms of Correlation and Explanation, the values are significantly higher when the template is consistent with the data than in the inconsistent combination. In contrast, the residual and Dispersion were significantly lower in consistent combinations than in inconsistent combinations. These results provide an important reference for the application of microstate sequence quality features in the identification of SCZ, as well as a beneficial reference for future research and clinical applications.

### 3.2 Microstate semantic analysis

**Figure 5:**
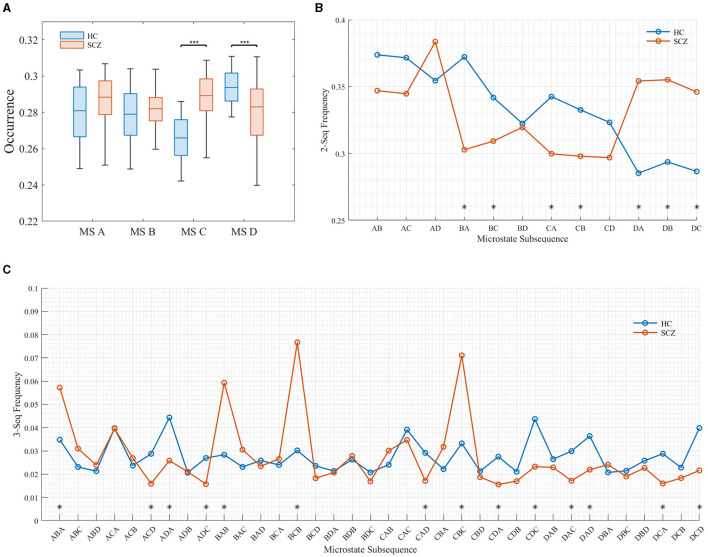
Frequency distribution of subsequences of different lengths. **(A)** Distribution of subsequences (*l* = 1). **(B)** Distribution of subsequences (*l* = 2). **(C)** Distribution of subsequences (*l* = 3).

This article focuses on the semantics expressed by the frequency of microstate subsequences. Figure 5 shows the frequency distribution of subsequences with different lengths.

It can be seen from the Figure 5 that when the length of the subsequence was 1 (as shown in Figure 5A, the frequency distribution of the subsequence with the length of 1 is equivalent to the microstate time parameter OPS), the frequency of microstate C and D in SCZ patients is significantly different from that of HC, in which the frequency of microstate C increases significantly and the frequency of state D decreases significantly. This is consistent with the results of da Cruz et al. (2020). When the subsequence length was 2, the frequency of BA, BC, DA, DB, and DC sequences in SCZ patients increased significantly, while the frequency of CA and CB sequences decreased significantly. When the subsequence length was 3, the frequency ABA, BAB, BCB, and CBC, was the highest in SCZ patients, which was much higher than that of healthy subjects. This suggests that there are specific subsequence patterns in the EEG signals of SCZ patients, and these subsequences express the semantics of the disease in SCZ patients.

It has been shown that microstate A is associated with phonological processing, microstate B with attention, microstate C with vigilance, and microstate D with visual processing (Britz et al., 2010). Thus, these high-frequency patterns of subsequences may reflect abnormalities in phonological processing, attention, and vigilance in SCZ patients. These abnormalities may be related to cognitive and affective disorders common to SCZ patients, such as attention deficit, impaired working memory, and emotional instability. In addition, these subsequence patterns may provide important clues for the diagnosis and treatment of SCZ patients. By analyzing the occurrence of these subsequence patterns in microstates, it can help clinicians diagnose SCZ patients more accurately.

### 3.3 Classification performances

#### 3.3.1 Microstate feature extraction

According to the experimental results in the previous section, a total of 39-dimensional microstate features were extracted as inputs to the classifier in this paper, including 16-dimensional quality features, 21-dimensional semantic features, and 12-dimensional traditional temporal features, and the feature distribution is shown in Table 2.

#### 3.3.2 The duration of EEG

This section focuses on the effect of microstate sequence length on SCZ recognition. For this purpose, the study introduces a sliding window approach, in which the original EEG data is segmented into EEG segments of length T, each with the same label as the original data, by setting a sliding window with window length T and window move 0. The goal of this approach is to systematically explore the effects of different microstate sequence lengths on the SCZ classification task. The optimal window length can be determined to improve the classification performance of SCZ. In addition, the application of the sliding window helps to increase the number of samples in the training and testing set, which is conducive to training the classifier more adequately. Specifically, the length of the sliding window ranges from 1 to 30 s, 30 datasets of different lengths were constructed at intervals of every 1 s, and 10-fold cross-validation was applied to each dataset to divide the training and testing sets. This approach can synthesize the classification performance under different window lengths, while effectively increasing the data samples to improve the performance of the classifier.

**Figure 6:**
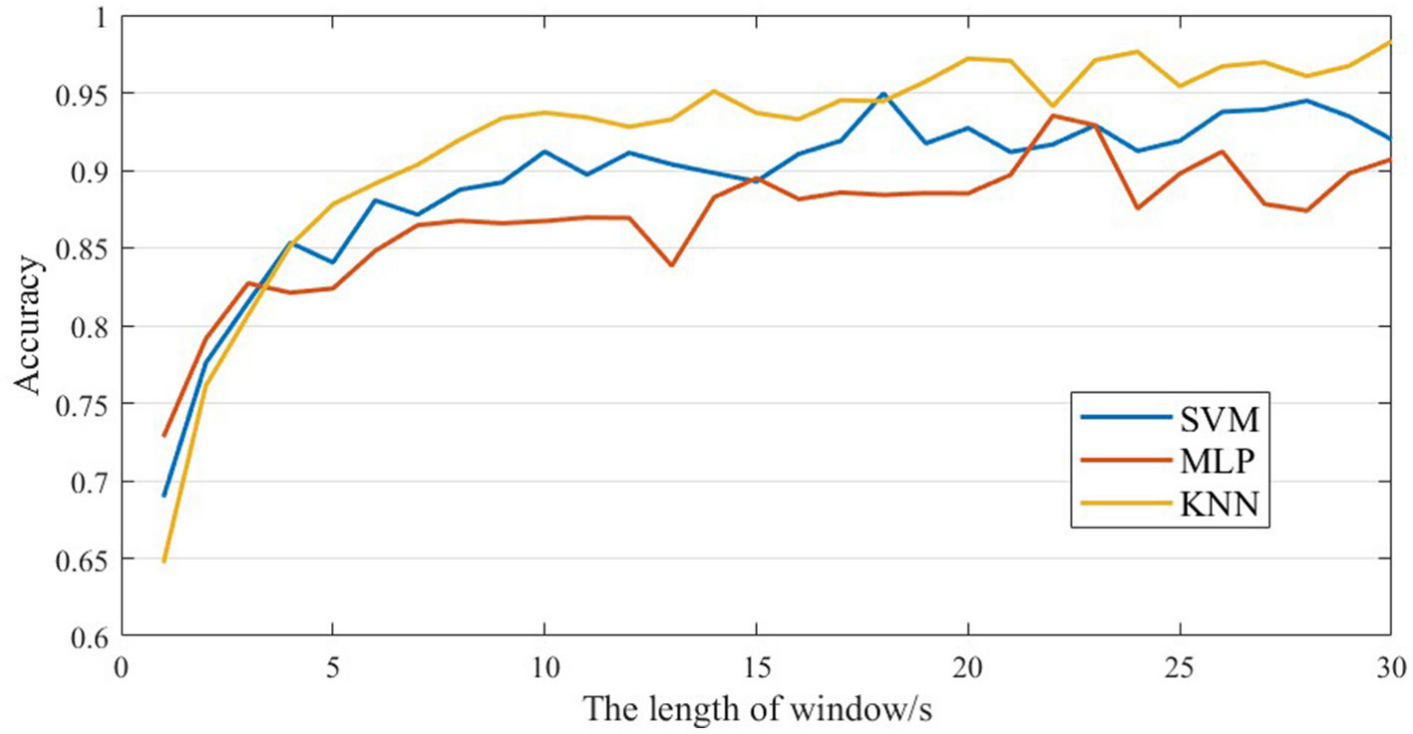
Correspondence plot between recognition accuracy and EEG length.

The samples are classified and validated using three classifiers, Support Vector Machine (SVM), *K*-Nearest Neighbor (KNN) and Multilayer Perceptron (MLP), the hyperparameters of the three classifiers are shown in Table 3 and the recognition results are shown in Figure 6.

**Table 3:** Classifier parameters.

**Classifier**	**Parameters**
SVM	Kernel: Gauss Kernel Scale: 6
KNN	Distance: Euclidean Number Neighbors: 10
MLP	Layers: 42*32*16*2 Activation function: Tanh

The experimental results show that at EEG segment lengths lower than 20 s, the recognition accuracies of SCZ all grow with the growth of EEG segment lengths, and the highest recognition rate of 97.2% is reached at 20s. When the EEG segment length is >20 s, the classification performance of the classifier falls into a bottleneck and starts to oscillate. In this paper, we analyze the reasons for the bottleneck and oscillations as follows: (1) The microstate sequence constructed when the EEG segment is 20 s already contains enough SCZ disease information, and the newly added EEG segment will not lead to the increase of SCZ disease information, but instead introduces additional noise and error. (2) Since the sliding windows used in this paper for EEG segmentation do not overlap, the total number of samples decreases as the length of the EEG segment increases (the total number of samples is 1,430 when the length of the EEG segment is 20 s), and the classifier is not sufficient to learn the features of the class from the existing samples, which leads to a decrease in the accuracy rate.

#### 3.3.3 Classification performances

Based on the experimental results, we found that the highest recognition accuracy of 97.2% is achieved when the EEG segment length is 20 s using KNN classifier. Compared with the published results, the proposed two-template microstate modeling analysis of schizophrenia diagnostic indicators in the identification of SCZ achieved the best results. The comparison results are shown in Table 4. Compared with the previous EMD decomposition (Siuly et al., 2020), signal energy or frequency analysis methods (Devia et al., 2019; Akbari et al., 2021), the micro-state method adopted in this paper is significantly improved. Compared with the existing microstate analysis techniques (Baradits et al., 2020; Kim et al., 2021), the sensitivity of the microstate sequence to the template is used to greatly enhance the recognition of SCZ. The experimental results fully demonstrate the effectiveness of the proposed indicators, which provides an effective basis for the clinical diagnosis of schizophrenia and the realization of intelligent diagnosis.

**Table 4:** The SCZ recognition accuracy of different features and classifier.

**Studies**	**Feature sets**	**Classifier**	**Accuracy**
Devia et al. (2019)	EEG activity	Linear discriminant analysis	71.00%
Baradits et al. (2020)	Microstate temporal parameters	Machine learning model	82.70%
Siuly et al. (2020)	EMD features	Ensemble bagged tree	89.60%
Akbari et al. (2021)	Phase space dynamic	*K*-nearest neighbor	94.80%
Kim et al. (2021)	Microstate temporal parameters	Support vector machine	75.60%
Lillo et al. (2022)	Microstate and microstate features	Convolutional neural networks	93.00%
Chen X. et al. (2023)	Linear and non-linear measures	Support vector machine	89.00%
This paper		*K*-nearest neighbor	97.20%

Further, this paper analyzes the sensitivity and specificity of the method proposed herein when the EEG fragment length is 20 s. Sensitivity is calculated by comparing the number of persons correctly identified as having a condition in a test population with the true number of individuals who have the condition in the same test population. Specificity is calculated by comparing the number of individuals correctly identified as not having a condition in a test population with the true number of individuals who do not have the condition in the same population. The sensitivity of the model was calculated to be 97.1% and the specificity was 97.3%. The experimental results indicate that the model trained using the microstate features proposed in this paper performs well in the recognition task of schizophrenia, and is able to distinguish between positive and negative cases effectively.

Finally, this study considers the generalization of the proposed method to cross-subject problems. In the previous classification task, we used a 10-fold cross-validation approach where the data from all subjects were mixed and randomly disrupted to verify the recognition accuracy. In order to more fully assess the generalizability of the method proposed in this paper, we used a leave-one-out method for validating the performance of the method in cross-subject schizophrenia recognition. The dataset used in this paper contains 14 schizophrenic patients and 14 normal subjects. In each test of the leave-one-out method, one patient's data and one normal person's data were selected as the test set, while the remaining 26 subjects' data were used as the training set, and this process was repeated 14 times. The final recognition results are shown in Table 5. This approach helps to assess the applicability of the proposed method across subjects and provides sufficient validation for its generalizability.

**Table 5:** The performances of cross subject classification.

**SCZ index**	**HC index**	**Accuracy**	**Sensitivity**	**Specificity**
1	1	97.9%	97.9%	97.9%
2	2	94.8%	94.1%	95.6%
3	3	98.0%	98.1%	97.9%
4	4	94.5%	93.2%	95.9%
5	5	95.9%	96.3%	95.4%
6	6	99.0%	98.4%	99.7%
7	7	97.1%	96.7%	97.6%
8	8	96.0%	95.7%	96.3%
9	9	96.1%	96.3%	95.9%
10	10	94.9%	94.8%	95.1%
11	11	96.8%	96.8%	96.8%
12	12	96.0%	96.8%	95.1%
13	13	94.0%	94.0%	94.0%
14	14	97.9%	97.9%	97.8%
Average		96.4%	96.2%	96.5%

The average recognition rate of SCZ across subjects reached 96.4%. This result suggested that the dual-template microstate analysis method has excellent generalizability across subjects. The method does not depend on specific individuals or subgroups, but has broader applicability. This has important implications for practical clinical applications, research and diagnosis of SCZ. In addition, the results also emphasize the importance of resting-state microstates as potential biomarkers or shared features of SCZ. Although schizophrenia typically exhibits a high degree of individual variability, resting-state microstates show a relatively high degree of stability.

#### 3.3.4 Ablation experiment

In this study, seven types of microstate features totaling 39 dimensions were extracted for classification analysis, and to further verify the effectiveness of the proposed features, this section uses feature importance to evaluate the importance of each feature in SCZ recognition. The computational process is as follows: Pre-train a KNN classifier using all features. Then set a certain type of microstate feature to be evaluated to 1. Finally, use the pre-trained classifier to classify the modified feature set. The amount of decay in the model performance represents the importance of that class of features.

The experimental results are shown in Table 6.

**Table 6:** Results of feature importance experiments.

**Types**	**Features**	**Dimensional**	**Accuracy**	**Importance**
Quality	Correlation	4	93.5%	3.7%
	xxxdummyxxx	Explained	4	91.5%	5.7%
	xxxdummyxxx	Residual	4	91.8%	5.4%
	xxxdummyxxx	Dispersion	4	91.9%	5.3%
Semantic	2seq frequency	7	90.7%	6.5%
	xxxdummyxxx	2seq frequency	14	91.0%	6.2%
Temporal	MD	4	94.4%	2.8%
	xxxdummyxxx	OPS	4	94.6%	2.6%
	xxxdummyxxx	TCR	4	95.5%	1.7%

The results of ablation experiments in this paper show that the extracted microstate features have a significant positive effect on the recognition of SCZ. Among them, semantic features have the highest percentage, which suggests that semantic features contribute greatly to understanding and classifying SCZ. It also strongly suggests that semantic sequences may carry information inherent to the brain states of SCZ patients. Second, the quality features of microstate sequences also occupy an important position, which implies that the dual-template approach proposed in this paper is reasonable and reliable. This approach extracts the quality features of microstate sequences based on the principle of template data consistency to effectively distinguish SCZ patients from healthy individuals. In contrast, traditional temporal features, such as the frequency, duration and percentage of microstates, are less important in SCZ identification. The results of the feature importance experiments validate the effectiveness of the features proposed in this paper, especially the semantic features and the quality features of microstate sequences, which have potential applications in SCZ recognition and research. These results provide an important basis for a deeper understanding of the brain mechanisms of SCZ and for improving its diagnosis.

The results of the classification experiments fully demonstrated that the microstate sequences constructed based on the resting-state EEG of schizophrenic populations carry relevant information about schizophrenic symptoms, which can be used for further schizophrenia-related research in academics, and can be used as biomarkers for effective detection of schizophrenic states in engineering.

## 4 Discussion

This study discusses the role of EEG microstates in the classification of SCZ. In contrast to conventional microstate features, we introduce two novel features: microstate semantic features and microstate quality features. The classification results suggest that the microstate features presented in this paper aid in effectively distinguishing between individuals with SCZ and healthy (control) subjects, yielding higher classification accuracy. This section will specifically focus on elucidating the microstate differences observed between individuals with SCZ and healthy subjects.

### 4.1 The difference in microstate template

In this paper, the spatial correlations of the two microstate templates were first analyzed in detail using the Pearson product-moment correlation coefficient. The results showed that there were significant differences between the SCZ patients and the HC in terms of microstate B and microstate D, with correlations of 0.68 and 0.86, respectively. Specifically, in microstate B, the activation of brain regions was mainly located in the left frontal lobe and the left temporal lobe for healthy subjects, whereas left temporal lobe activation was significantly lower for SCZ patients. In microstate D, the activation of brain regions in healthy subjects was mainly concentrated in the frontal and parietal lobes, and exhibits lower left and right brain laterality. On the contrary, the activation of brain regions in SCZ patients showed significant lateralization, with the activation in the right hemisphere being significantly higher than that in the left hemisphere. It has been shown that microstate B is associated with attention and microstate D with visual processing (Britz et al., 2010). The differences in these microstate templates further highlight the significant changes in the overall brain working mode of SCZ patients, and indicate that the disease information of SCZ is reflected in the microstate. The topography of type B and D showed a significant difference between the two groups which is consistent with previous studies on SCZ studies. Microstate B and D were reported by several studies and found to be associated with positive symptoms (Lehmann et al., 2005; Nishida et al., 2013; Kim et al., 2021).

Microstate alterations observed in SCZ seem to reflect deteriorated connectivity, decreased functional organization, or increased noise in brain processes that have been hypothesized as neurophysiological bases for SCZ symptomatology (Lehmann et al., 2005). Britz et al. (2010) discovered that microstate B exhibited a correlation with negative BOLD activation in the bilateral occipital cortex, while microstate D was associated with negative BOLD activation in the right-lateralized dorsal and ventral areas of the frontal and parietal cortices. According to Milz et al. (2016), each microstate may correspond to a specific function network, which corresponds to the auditory network, visual network, salience network, and attention network of the brain, respectively. Abnormalities in EEG microstates observed in SCZ imply a disruption in normal network activities underlying the pathogenesis of the disease. Altered microstate characteristics may indicate changes in the propensity for activation of specific neural assemblies.

We found no public study which discussed the relationships between microstate differences and molecular changes in SCZ. Microstate analysis mainly focuses on the macro level of brain activity, while molecular changes occur at the micro level, lacking direct connection bridges. Nevertheless, we can still speculate that there may be a correlation between molecular changes and disrupted microstate characteristics. *In vivo* imaging of the dopamine system has consistently identified elevated striatal dopamine synthesis and release capacity in SCZ (McCutcheon et al., 2020). Disruption in the glutamatergic system due to NMDA receptor alteration, which has been shown in schizophrenia (Balu, 2016). Buck et al. (2022) proposed that disrupting DA-glutamate circuitry between dopamine and glutamate, particularly in the striatum and forebrain, is the pathophysiology that leads to SCZ. Molecular changes may result in inefficient cortical network synchronization, yielding different characteristic for the corresponding microstates. However, empirical studies are needed to gain insight into the connection between molecular alterations and microstates disturbances.

### 4.2 The difference in microstate sequence features

**Figure 7:**
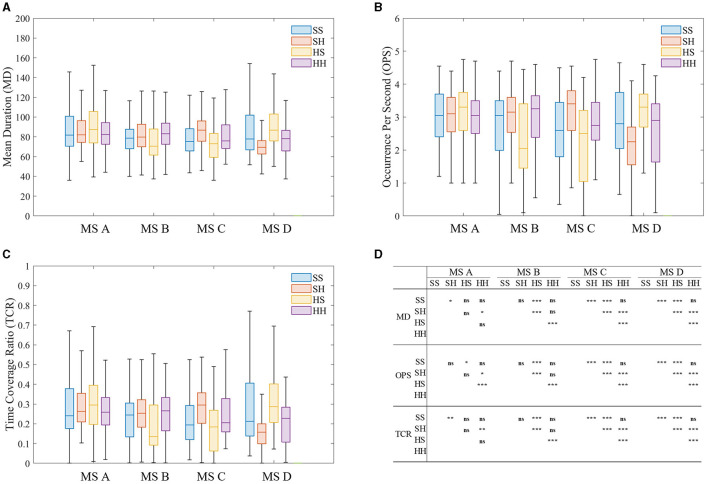
Results of microstate Sequence feature analysis of four microstate sequences. **(A)** Mean duration (MD). **(B)** Occurrence per second (OPS). **(C)** Time coverage ratio (TCR). **(D)** The results of Wilcoxon rank sum test. *p < 0.05, **p < 0.01, ***p < 0.001.

The microstate sequence MD, OPS, TCR, and TP features were extracted and statistically analyzed. The results are shown in Figure 7. As can be seen from Figure 7, the differences of MS-seq features mainly focus on microstate C and microstate D no matter which microstate template was used. MD, OPS, and TCR of microstate C increased significantly, while MD, OPS, and TCR of microstate D decreased significantly. The time course of the microstates contains important information about the underlying neural generator (Khanna et al., 2015). In SCZ patients, the stability, tendency, intensity, or coordination of neural components are altered.

This result is consistent with previous studies, indicating that microstate features are stable and suitable for classification of SCZ. A large body of prior literature found increased parameters of class C and decreased parameters of class D in patients with SCZ compared with healthy controls (Britz et al., 2010; Amad et al., 2014; Milz et al., 2016). Combining the results from the previous section, it is demonstrated that the temporal characteristics of microstates are closely related to the topological structure characteristics. Microstate class C was associated with the salience network, which functions to identify the most relevant stimulus from internal and external inputs to guide appropriate actions (Thijssen et al., 2015). Therefore, altered parameters of class C in SCZ may be closely related to the clinical manifestations of SCZ patients who have difficulty distinguishing between the inner world and the outside world. Similarly, microstate class D is related to the frontoparietal attention network. The change of class D in SCZ may reflect impaired cognitive functions involving attentional processes.

When using different templates to model the same type of EEG signal, we found that MD, OPS and TCR of microstate B were more sensitive to microstate templates and EEG data types, that is, only when microstate modeling was performed on healthy EEG data, the above characteristics showed differences, while the EEG data of SCZ patients showed no differences. In contrast, MD and TCR of microstate A showed the opposite behavior and were only sensitive to the microstate sequence of SCZ patients.

### 4.3 The difference in microstate semantic features

In Section 3.2, it is noted that at a subsequence length of 2 (i.e., two microstates appearing in pairs), there is a significant increase in the probability of occurrence of the BA, BC, DA, DB, and DC sequences in SCZ patients. Conversely, the probability of occurrence of the CA and CB sequences decreases significantly. Furthermore, at a subsequence length of 3 (i.e., three microstates appearing simultaneously in a fixed order), SCZ patients exhibit the highest frequency of the ABA, BAB, BCB, and CBC subsequences, surpassing those observed in healthy subjects. These findings suggest the presence of specific subsequence patterns in the EEG signals of SCZ patients. The heightened occurrence of these subsequence patterns may, therefore, reflect abnormalities in speech processing, attention, and vigilance in individuals with SCZ.

Currently, research on microstates has predominantly concentrated on the neural representations of individual microstates, with limited attention dedicated to microstate sequences. In this paper, we identify several fixed microstate sequences in patients that exhibit significant differences compared to healthy subjects. As previously discussed, the topological structures of microstates B and D exhibit substantial alterations in SCZ patients compared to healthy individuals. These changes disrupt the transition between states. Baradits et al. (2020) found the transition from one state to another may represent the sequence of networks that constitute large-scale brain networks. Disturbance in such a structure of network operations may result in disconnection between brain networks, which thereby leads to dysfunctional behavior.

When analyzing subsequences with a length of 3, it is essential to consider them as a unified entity. Currently, there is limited research exploring the correlation between microstate subsequences and the pathology of SCZ. From Figure 7, it is evident that abnormal subsequence states can be broadly categorized into two types: first, those where subjects struggle to return to their original state after a transition, and second, transitions between states ACDs. The first type of abnormality suggests that SCZ patients face challenges in reverting to their original state post-transition, potentially linking to clinical symptoms such as disjointed thinking, incoherent speech, or compulsive thoughts commonly observed in SCZ diagnosis. These abnormal subsequences may directly correspond to these clinical manifestations. The second type of anomaly primarily occurs during transitions between states ACD. According to research by Milz et al., microstate A represents the auditory network of the brain. Therefore, abnormalities in state ACD transitions may be closely associated with verbal hallucinations experienced by SCZ patients.

These subsequence patterns may offer valuable insights for the diagnosis and treatment of SCZ patients. By analyzing the occurrence of these subsequence patterns in microstates, clinicians may be able to diagnose SCZ patients with greater accuracy.

### 4.4 Limitation

Some study limitations must also be discussed.

First, this study used publicly available data instead of data obtained from cohort studies, and the number of subjects was small. Although age and gender were matched, it was difficult to obtain enough data to represent the general population. Thus, the results of this study should be interpreted carefully.

Secondly, as the dataset used in this study is public, the analysis did not include an examination of patients' clinical manifestations alongside their microstate manifestations. In our future research, we intend to collaborate with hospitals and other organizations to undertake a more in-depth exploration of the relationship between clinical performance and microstates in SCZ.

Finally, due to the constraints posed by the data length, the analysis of the semantic features of microstates in this paper was limited to sequences of length 3. In subsequent studies, we aspire to extend this investigation to obtain microstate markers that offer a more comprehensive characterization of schizophrenia.

## 5 Conclusion

In this study, we propose a method for investigating the brain activity of patients with schizophrenia based on the microstate semantic modeling method. This method introduces the concept of microstate semantic features, decomposes microstate sequences into subsequences of varying lengths, compares their statistical features, and successfully extracts subsequences with specific semantics. These specific sequences characterize the distinctive brain activity patterns of SCZ. Additionally, a dual-template microstate construction strategy is employed to define the quality features of microstate sequences across four dimensions: relevance, explanation, residual, and scatter. The quality features and semantic features of microstate sequences were tested on public datasets for SCZ recognition, achieving an accuracy rate of 97.2%. Furthermore, cross-subject experimental validation is conducted, demonstrating that the method achieves a recognition rate of 96.4% between different subjects.

The studies show thatmicrostate sequences are a valid electrophysiological marker for the identification of psychiatric classified disorders. However, we also need to realize that to truly apply this technique to clinical diagnosis, more studies of the same kind are needed to extend the research and to conduct more in-depth analytical studies to better understand the relationship between EEG microstate sequences and psychiatric classified disorders in order to provide better support for clinical practic.

## Data availability statement

The original contributions presented in the study are included in the article/supplementary material, further inquiries can be directed to the corresponding author.

## Ethics statement

The studies involving humans were approved by the Ethics Committee of the Institute of Psychiatry and Neurology in Warsaw. The studies were conducted in accordance with the local legislation and institutional requirements. Written informed consent for participation was not required from the participants or the participants' legal guardians/next of kin in accordance with the national legislation and institutional requirements. Written informed consent was obtained from the individual(s) for the publication of any potentially identifiable images or data included in this article.

## Author contributions

HoL: Methodology, Validation, Visualization, Writing – original draft, Writing – review & editing. CW: Conceptualization, Investigation, Writing – review & editing. LM: Investigation, Writing – review & editing. CX: Writing – review & editing. HaL: Funding acquisition, Methodology, Writing – review & editing.
